# Cellulose Nanocrystals and Rice Husk Surface Functionalization Induced by Infrared Thermal Activation

**DOI:** 10.1002/cssc.202500164

**Published:** 2025-03-27

**Authors:** Rosarita D'Orsi, Chiara Danielli, Mariachiara Spennato, Elisa Guazzelli, Elisa Martinelli, Fioretta Asaro, Lucia Gardossi, Alessandra Operamolla

**Affiliations:** ^1^ Dipartimento di Chimica e Chimica Industriale Università di Pisa via Giuseppe Moruzzi 13 56124 Pisa Italy; ^2^ Consorzio Interuniversitario Nazionale di ricerca in Metodologie e Processi Innovativi di Sintesi C.I.N.M.P.I.S. 56124 Pisa Italy; ^3^ Department of Chemical and Pharmaceutical Sciences University of Trieste Via L. Giorgieri 1 34127 Trieste Italy; ^4^ Interuniversity Consortium of Chemical Reactivity and Catalysis (CIRCC) ViaCelso Ulpiani 27 I-70126 Bari Italy

**Keywords:** Cellulose nanocrystals, rice husk, solvent-less functionalization, nuclear magnetic resonance, organic synthesis, hydrophobization, infrared thermal activation

## Abstract

Infrared thermal activation (IRTA) is considered an efficient approach to accelerate reaction rates. The manuscript reports the first example of application of IRTA to achieve surface functionalization of cellulose nanocrystals (CNCs) under solvent‐less conditions with epoxidized linoleic acid (ELA), synthesized by enzymatic approach using CaLB (lipase B from *Candida antarctica*) and H_2_O_2_. The final goal is to enhance the hydrophobicity of cellulosic surfaces of bio‐based materials, with potential application in the coating industry. With the approach proposed in this paper, we achieve a degree of substitution of 0.09 of CNCs. The reaction is extended to delignified rice husk (d‐RH), a largely available agro‐waste and a cost‐effective cellulose‐rich biomass. Solid‐state cross polarization magic angle spinning (CP MAS) ^13^C nuclear magnetic resonance (NMR) analyses, liquid state ^1^H‐NMR, attenuated total reflectance Fourier transform infrared spectroscopy (ATR‐FTIR), field emission scanning electron microscopy (FE‐SEM) and X‐ray diffractometry (XRD) data support a fine structural characterization of both functionalized CNCs and d‐RH to assess the effectiveness of the strategy used and the characteristics of the materials. Water contact angle measurements confirm the changed surface chemistry and the occurrence of hydrophobization on CNCs and d‐RH, revealing surfaces with modified properties and stable water contact angle of ∼40° and ∼60°, respectively. This efficient and sustainable method can have potential application in industrial‐scale environments to change the properties of ligno‐cellulosic biomass and bio‐based materials in general.

## Graphical abstract

Cellulose nanocrystals are used as a benchmark to demonstrate the functionalization of cellulose with epoxidized linoleic acid under infrared assisted thermal activation (IRTA) conditions. The approach is easily extended to delignified rice husk.

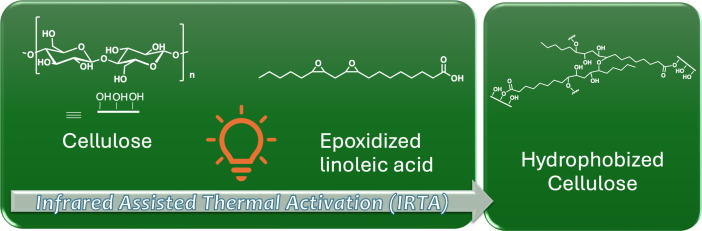

## 1 Introduction

The use of organic polymers in packaging,^[1]^ water‐repellent/self‐cleaning materials,^[2]^ coatings,^[3]^ oil and water separation,^[4]^ presents notable environmental challenges. Plastic usage contributes significantly to marine and terrestrial pollution, posing threats to wildlife and ecosystems.[^5^, ^6^] Moreover, the production of plastics often entails the consumption of fossil fuels and emits greenhouse gases, exacerbating climate change. It is imperative to transition towards more benign choices to alleviate these environmental pressures. Various sustainable alternatives have been proposed, including bio‐based polyesters,^[7]^ polyamides,^[8]^ cellulose and nanocellulose,^[9]^ other polysaccharide derivatives,^[10]^ and lignin.^[11]^ A particularly promising avenue involves utilizing waste from agricultural biomass, such as milled microparticles from rice husks (RH), which can be a cost‐effective alternative to nanocellulose. These approaches present opportunities for developing new, high‐value products. For example, hydrophobized rice husk could be tested in composite materials for furniture, automotive parts, or as a filler in biodegradable plastics, offering both functional and environmental advantages.^[12]^ For this reason, achieving an efficient and sustainable method to tailor the surface chemistry and properties of bio‐based materials, including nanocellulose and rice husk, is highly desirable.^[9]^

In this study, we introduce an innovative approach utilizing infrared thermal activation (IRTA)^[13]^ for the surface functionalization of nanocellulose and rice husk with epoxidated linoleic acid. Our investigation begins with neutral cellulose nanocrystals (N_CNCs) as the benchmark cellulose‐rich material to study surface functionalization chemistry.[^14^, ^15^] Subsequently, we extend our methodology to rice husk microparticles, showcasing the efficacy of our proposed technology. The functionalization reaction is designed not only to modulate the hydrophobicity and surface chemistry of the initial material but also to illustrate its potential application in industrial‐scale environments.

Infrared (IR) heating systems offer scalability, enabling adaptation to diverse industrial settings, while providing precise control over temperature profiles without necessitating intermediary heat transfer mechanisms. Hence, this study features the promising perspective of utilizing IR thermal activation for large‐scale surface functionalization. IR activation of chemical reactions is a new frontier of green chemistry, which complies with the 6^th^ principle by minimizing the energy required to trigger reactivity.^[13]^ Furthermore, IR thermal activation can accelerate reaction rates improving process efficiency.^[16]^ So far, IR thermal activation has been used on biomass as a pretreatment to facilitate extraction of natural products like *p*‐coumaric acid, vanillin or other shikimic acid derivatives, sugars or polyphenols.[^17^, ^18^, ^19^, ^20^] Its use on cellulose, instead, is limited to an experimental setup, proposed in 1988, for the artificial aging of historical textiles, based on the use of a CO_2_ laser operating at 10.6 μm wavelength (corresponding to a photon energy well below that required to induce chemical reactions), for the selective thermal degradation of small areas of large historical objects.^[21]^

So far, no reaction has been reported on pure cellulose or nanocellulose triggered by IR thermal heating. Considering the aforementioned factors, we opted to explore, for the first time, the surface reactivity of cellulose nanocrystals in IR‐activated chemical reactions. Our objective was to develop a product with promising applications in materials technology. Thus, we focused on surface hydrophobization, with the aim of potentially extending its applicability to other accessible cellulose‐rich materials, such as rice husk. This investigation aims to broaden the scope of functionalization techniques for cellulose‐based materials, opening avenues for innovative applications in various fields.

## 2 Results and Discussion

### 2.1 Preliminary Investigation on Cellulose Nanocrystals (CNCs)

Cellulose nanocrystals (CNCs) represent an interesting benchmark to study the surface functionalization of cellulose nano – or micro‐structures,^[22]^ since they are highly crystalline, monodisperse, and it is possible to apply on them liquid state ^1^H NMR spectroscopy to study their surface functionalization.[^14^, ^23^] First, we needed to understand the feasibility of the application of IR‐triggered reactivity to CNCs. Therefore, we studied the effects of exposure to IR irradiation at ~120 °C for 15 minutes on both sulfated and neutral cellulose nanocrystals (S_CNCs and N_CNCs, respectively). Sulfate groups in S_CNCs are randomly present on the primary hydroxyl groups on the surface glucopyranose units as a consequence of the surface esterification with sulfuric acid, used to produce this typology of nanomaterials.^[24]^ Previous studies had already shown the lower thermal stability of this form of nanocellulose.[^15^, ^24^] The irradiation of S_CNCs under IR light, without any catalyst, caused darkening of the starting material, suggesting their decomposition, quite probably auto‐catalyzed by the surface sulfate groups.^[25]^ N_CNCs, nanocrystals composed of pure cellulose, instead, were unaffected by the same treatment for 15 minutes. Therefore, for the following experiments, we selected as reference materials N_CNCs prepared from Avicel by HCl hydrolysis, which were compatible with IR thermal treatment.

**Scheme 1:**
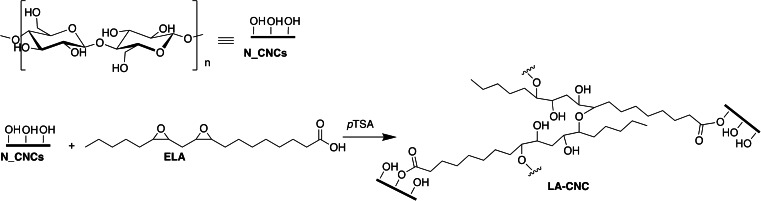
Model of the most probable acid catalyzed reaction occurring between CNCs and ELA under solvent‐free conditions with IR‐thermal activation. Oxirane ring opening can occur by reaction with pending carboxyl groups, pending ‐OH of cellulose, or ‐OH groups generated by another ring opening reaction.

 A ring‐opening reaction of epoxy‐derivatives of unsaturated fatty acids was chosen as a model functionalization reaction. Previously, a similar reaction was demonstrated under solvent‐free conditions between sorbitol and epoxidated soybean oil.^[26]^ The reaction, triggered by the temperature in the reference paper, appeared ideal to start investigating the behavior of nanocellulose in place of sorbitol, under solvent‐free and IR‐thermally activated reaction conditions. Sorbitol represents an effective monomeric representative for cellulose: it has no available anomeric hydroxyls as cellulose, but it exhibits a high number of available alcoholic functions and insolubility in organic solvents as chloroform. As a reaction partner, we selected epoxidated linoleic acid (ELA), which has the potential to conduct to cross‐linked cellulose nanocrystals. ELA was sustainably synthesized through the solventless chemo‐enzymatic epoxidation of linoleic acid with CalB (lipase B from *Candida antarctica*).^[26]^ For comparison, and to demonstrate the feasibility of this strategy, we performed the solvent‐free reaction of ELA with sorbitol both in a nitrogen atmosphere with conventional heating and in air under IR‐thermal activation. During the reaction pre‐screening, the use of *p*‐toluenesulfonic acid (*p*TSA) as catalyst was preferred to sulfuric acid because the last generated darkening of the reaction mixture under irradiation. Indeed, the ease of dehydration and carbonization of sugars under irradiation by sulfuric acid is well known.^[27]^ NMR spectra on both products were acquired in CDCl_3_ (see Experimental Data, Figures S3–4). ^1^H NMR showed that the isolated products obtained by thermal heating and IR thermal activation are quite similar. In both cases the signals referring to linoleic acid molecular skeleton appeared broader. The signals of epoxydic protons disappeared,^[28]^ indicating that a polymerization occurred, and ring‐opening took place. The appearance of sorbitol signals indicated the presence of the sugar in the polymerized acid. Since pure sorbitol is soluble in d^6^‐DMSO, and insoluble in CDCl_3_, this is a sign that sorbitol has been covalently linked to the linoleic derivative. The ^13^C NMR spectrum, acquired on the product cross‐linked by *p*‐toluensulfonic acid catalyst, revealed the parallel esterification of the free carboxyl groups of ELA (Figure S5), through the appearance of signals at 173 and 174 ppm. The formation of an ester is supported by a literature example reporting IR assisted esterification of glycerol.^[29]^ This observation, paired to the capacity of ELA to self‐polymerize in the presence of acid, allowed us to infer that in the case of nanocellulose, the most probable covalent link induced between the surface of CNCs and ELA is an ester bond, whilst the more flexible ELA chain can self‐polymerize through ring opening reaction. Another possible cross‐linking mechanism involves the oxirane ring opening by the pending carboxyl groups of ELA. The corresponding hypothesized reaction scheme is shown in the Scheme 1.

The reaction between N_CNCs and ELA was performed under IR light thermal activation in an open vessel in air. The reaction was run for 15 minutes, and the reached temperature was ~120 °C. The product was washed with aqueous ammonia to remove excess ELA and *p*TSA. The product was isolated with a 62 % mass recovery with respect to the starting materials. Several characterizations were carried out on the isolated product, which was labeled **LA‐CNCs** (linoleic acid – cellulose nanocrystals).

**Figure 1:**
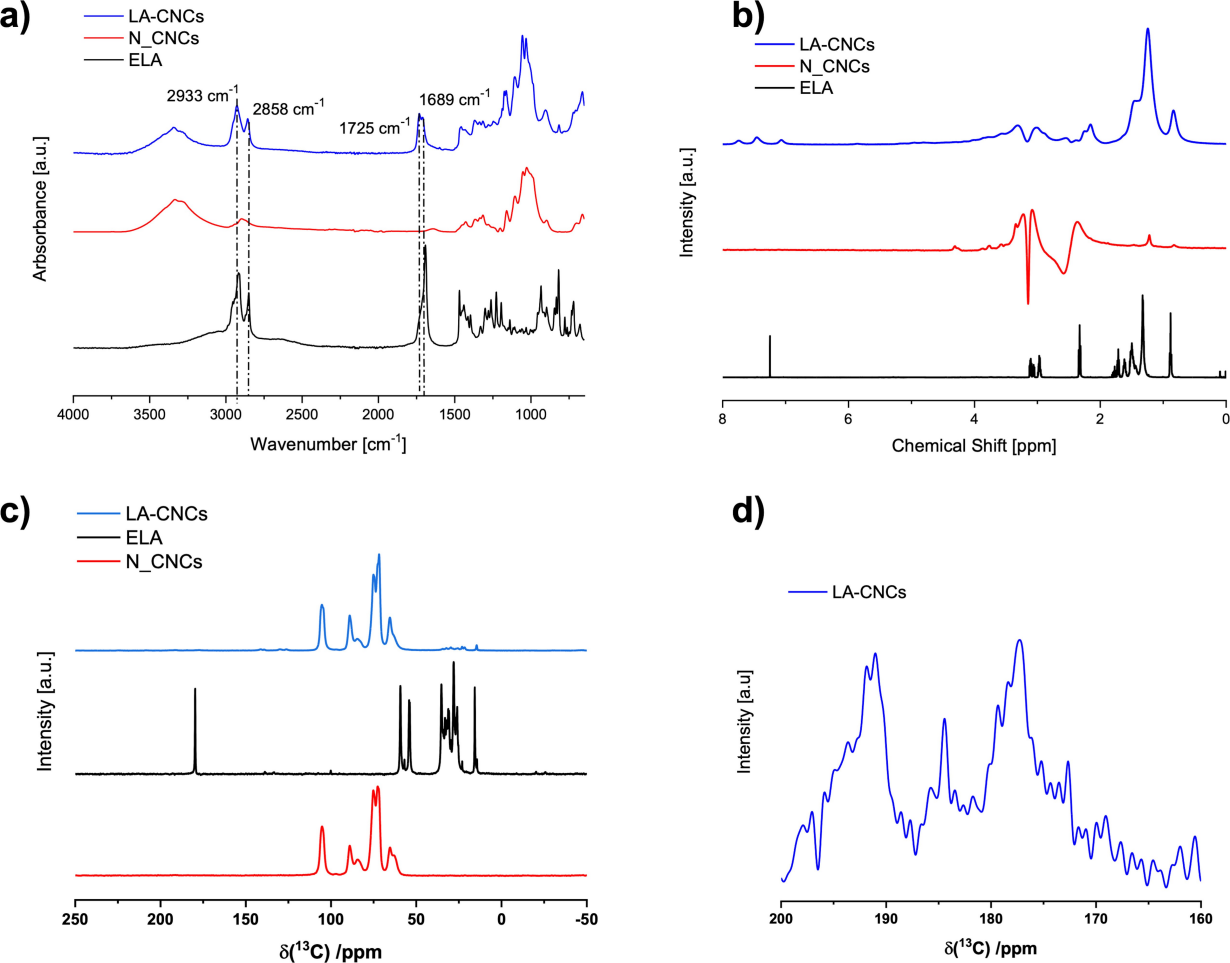
a) ATR‐FTIR spectra of ELA (black line), N_CNCs (red line), and LA‐CNCs (blue line) produced under *p*‐toluenesulfonic acid catalysis; b) liquid state ^1^H‐NMR spectra acquired in DMSO‐d_6_ of ELA (black line), N_CNCs (red line), and LA‐CNCs (blue line) applying a wet sequence for the suppression of solvent and water signals; c) solid state CP‐MAS ^13^C‐NMR spectra of ELA (black line), N_CNCs (red line), and LA‐CNCs (blue line); d) enlargement of the solid state CP‐MAS ^13^C‐NMR spectrum of LA‐CNCs in the carboxyl region.

The Figure 1 collects attenuated total reflectance Fourier transform infrared (ATR‐FTIR) spectra, liquid state and solid state nuclear magnetic resonance (NMR) spectra of ELA, N_CNCs and of the cross‐linked product, LA‐CNCs. LA‐CNCs ATR‐FTIR spectrum (Figure 1a, blue trace) showed the presence of specific signals at 1689 cm^−1^ and around 2858 and 2933 cm^−1^, related to the free carboxyl C=O and aliphatic C‐H stretching modes in linoleic acid. At the same time, the presence of cellulose is undoubtful as per the appearance of acetal signals at 1000–1100 cm^−1^ and of the stretching of the pending hydroxyl groups signal observed at ~3300 cm^−1^. The formation of ester bonds is confirmed by the appearance of a strong absorption band centered at 1725 cm^−1^, shifted with respect to the absorption band of ELA centered at 1689 cm^−1^.

^1^H NMR spectra of cellulose nanocrystals in d_6_‐DMSO colloidal dispersion are useful to get information about their more mobile surface structures.^[14]^ Spectra were recorded at 65 °C on ~20 mg mL^−1^ dispersions using a wet sequence to suppress the residual signals of water and DMSO. The ^1^H NMR spectrum of N_CNCs is reported in Figure 1b. The distortion of the baseline, due to the suppression of the signals at 3.11 and 2.46 ppm, is particularly evident in this spectrum for the low intensity of the signals from the surface groups of the nanocrystals composed of pure cellulose. The signals include the anomeric proton (H1) (4.37–4.21 ppm), the diastereotopic protons (H6) of the methylene C6‐OH group (two signals at 3.91–3.71 ppm), the signals of H3, H4, H5 (3.71–3.47 ppm) and the H2 signal (3.35 ppm). The rapid exchange with water originating from the nanocrystals themselves was considered responsible of exchanging with the surface hydroxyl groups of N_CNCs. Water is entrapped in the cellulose structure upon drying, as revealed by elemental analyses and ATR‐FTIR spectra (Figure 1a, absorption band centered at 1641 cm^−1^). After reaction with ELA, distinctive signals of linoleic acid appear in the ^1^H NMR spectrum of LA‐CNCs in the dispersed state: aliphatic signals at chemical shifts >0.8 ppm up to 3 ppm are clear in the spectrum of the functionalized product and indicate the co‐presence of the two molecular skeletons in the final product. Furthermore, aromatic signals arise in LA‐CNCs, proving the further reaction of cellulose nanocrystals with *p*TSA producing a *p*‐toluenesulfonic ester.

CP MAS ^13^C NMR solid state nuclear magnetic resonance spectra were acquired on the two samples to further demonstrate the success of the reaction and to estimate the crystallinity change of cellulose nanocrystals before and after the reaction. The low intensity of signals attributable to linoleic acid in the LA‐CNCs spectrum announced a low substitution degree achieved by the reaction. Nonetheless, ssNMR spectra were extremely useful to appreciate the formation of ester bonds from the linoleic acid. Indeed, the magnification of the specific region of the spectrum for LA‐CNCs, shown in Figure 1d, illustrates the clear appearance of ester functions in the cross‐linked product. Further information is given by the appearance of aliphatic signals from ELA between 0 and 40 ppm in the cross‐linked product, whilst the signals of the epoxydic carbons were not detected. The crystallinity index (CRI), estimated from the comparison of the peak areas for C4,[^30^, ^31^] increased from ~54 % to ~61 %. This pointed not only at a negligible degradation of the cellulose crystal structure during the IR thermal treatment, but eventually to an increase of crystallinity due to removal of amorphous regions from the nanocellulose structure during the washing steps.

Elemental analyses, reported in the Table 1 for N_CNCs and LA‐CNCs, allowed us to derive the raw formula for pristine and functionalized cellulose nanocrystals (please compare Supplementary Data for the calculation). The increase in nitrogen was attributed to ammonia absorbed by the sample during the work up, finalized at dissolving unreacted linoleic acid, whilst the increase in carbon was attributed to the linoleic acid and to the *p*TSA forming ester bonds with the nanocellulose. The sulfur percentage allowed us to differentiate between *p*TSA and LA content in the final product. According to these observations, we calculated a degree of substitution (DS) of 0.05 for *p*TSA covalently linked as ester and a DS of 0.09 for LA covalently linked to the nanocellulose. The low DSs found are in perfect agreement with the crystallinity index found in LA‐CNCs: indeed, to preserve cellulose nanocrystals crystallinity, it is necessary to perform topochemical functionalization in very mild conditions, yielding very low DS to respect the aggregation stability of cellulose chains.[^22^, ^32^, ^33^] Furthermore, the low degree of substitution with LA reflects the low intensity of its signals as seen from the solid state ^13^C NMR experiments.

**Table 1:** Compositional values, calculated degree of substitution and crystallinity indexes of **N_CNCs** and **LA‐CNCs**.

Sample	Elemental Analyses					DS_pTSA_^b^	DS_LA_^c^	CRI^d^
	C	H	N	S	O^a^			
N_CNCs	41.54	6.30	0.03	0.002	52.13	–	–	54 %
LA‐CNCs	47.96	7.12	0.29	0.76	43.87	~0.05	~0.09	61 %

^a^ Oxygen content determined by difference. ^b^ Degree of substitution with *p*‐toluenesulfonic acid (*p*TSA). ^c^ Degree of substitution with LA. ^b,c^ The two degrees of substitution for cellulose were calculated from elemental analyses, as described in the Supplementary Data. ^d^ Crystallinity index of cellulose, calculated from solid state CP MAS ^13^C NMR spectra after peak deconvolution with the software MestreNova.

**Figure 2:**
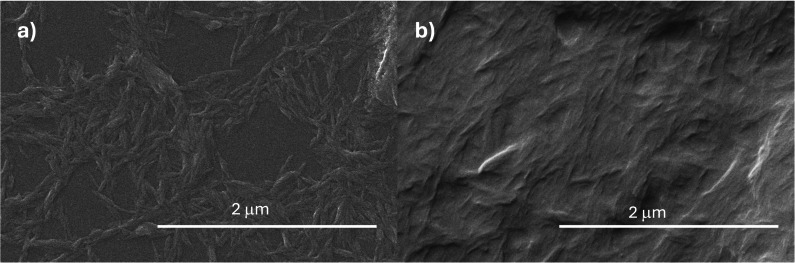
FE‐SEM micrographies of a) N_CNCs and b) LA‐CNCs. Samples were deposited on glass from a 1 mg L^−1^ DMSO suspension. Scale bar: 2 μm.

Field Emission Scanning Electron Microscopy (FE‐SEM) micrographies of N_CNCs and LA‐CNCs are displayed in the Figure 2. Each sample was deposited from a 1 mg L^−1^ DMSO suspension. The surface functionalization in LA‐CNCs dramatically affected the aggregation tendency of CNCs: whilst N_CNCs (Figure 2a) showed tendency to aggregate randomly, even if the DMSO is a solvent chosen *ad hoc* to interfere with the inter‐crystal hydrogen bonds, and the micrography hardly allows to recognize individual nanocrystals, LA‐CNCs (Figure 2b) showed a strong film‐forming tendency. In LA‐CNCs, cellulose nanocrystals appear dispersed in an organic matrix, produced by the linoleic acid, homogeneously mixed to CNCs as a result of the occurred chemical reaction. In LA‐CNCs, nanocrystals appear interacting by the long side. The morphology strongly supports the cross‐linked nature of LA‐CNCs product.2

**Figure 3:**
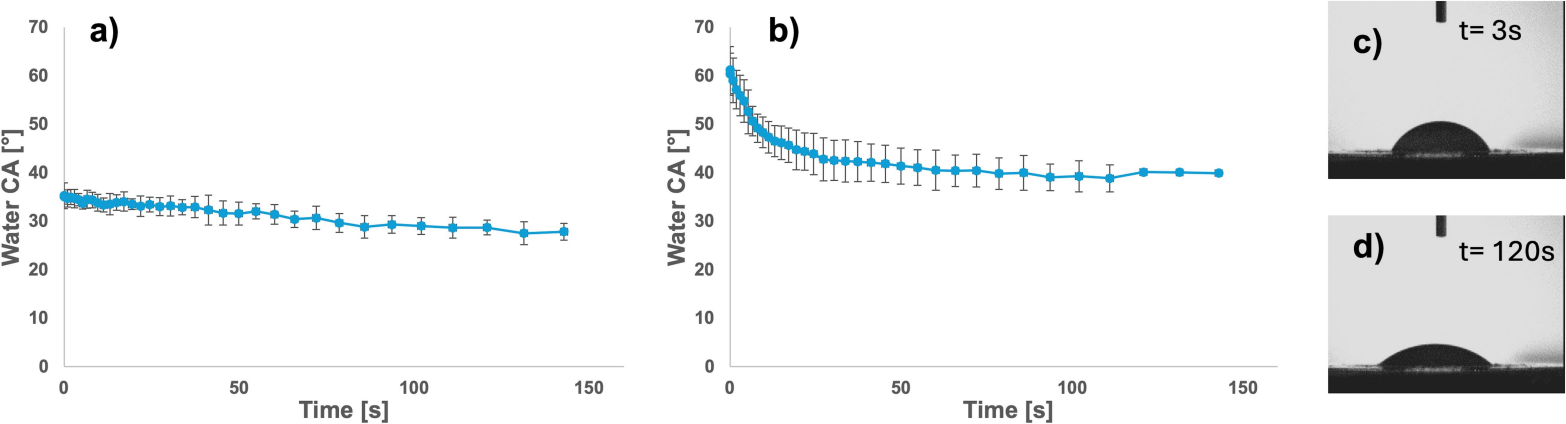
Water contact angle as a function of time up to 120 s for: a) N_CNCs and b) LA‐CNCs thin films deposited on glass. Digital photo of the water droplet deposited on LA‐CNCs c) at t= 3 s and d) at t= 120s, showing the stability achieved by the system.

Finally, to demonstrate the changed surface chemistry and energy of CNCs after the functionalization, we carried out static water contact angle measurement on thin films of N_CNCs and LA‐CNCs drop cast on glass from DMSO. In the case of N_CNCs thin films, the water contact angle could not be detected. The water drop penetrated progressively into the nanocellulose film during the first few seconds from the deposition. The water contact angle change over time on N_CNCs films is reported in the Figure 3a. This demonstrated the easy uptake of water into the pristine N_CNCs thin films, pointing at a complete wettability. The primary effect is the swelling and overall weakening of the film compactness which prevents its application as water barrier, for instance. Hexadecane showed  complete wettability on N_CNCs (Figure S6). The disorder of the deposited film and the amphiphilic nature of cellulose nanocrystals composed of allomorph I_β_ are usually responsible of the wettability of their thin films in water and organic solvents, as well, with changes depending on the conditions used to prepare nanocrystals thin films.^[34]^ Conversely, the contact angle of distilled water on the surface of the LA‐CNCs thin film was equal to 60° as deposited, evolving in ~120 s to the value of 40° (Figure 3b). After this time, the water drop was stable on the LA‐CNCs thin film. The measurements were repeatable, with the drop being wiped off the film and redeposited on it, and showing always the same behavior (compare Figure S7). This proved the change in the grade of wettability due to the successful functionalization. LA‐CNCs films displayed complete wettability from hexadecane, as expected.

### 2.2 Extension of the Functionalization Procedure to Delignified Rice Husk

Cellulose nanocrystals are a nice opportunity to test the reactivity of cellulose and may be interesting for high value applications. However, the current cost of nanocellulose (~1.5 $ g^−1^ for sulfated CNCs, whilst neutral CNCs are presently not commercially valued at present)[^35^, ^36^] preventstheir application in large volume in low‐cost products. The highly efficient enzymatic synthesis of neutral cellulose nanocrystals could solve the cost problem,^[37]^ but the low dispersibility of neutral CNCs represents an obstacle to many applications.^[38]^

The synthetic approach applied to the neutral cellulose nanocrystals was then extended to delignified rice husk (d‐RH), a cost effective and renewable waste product from rice cultivations.^[39]^ Even if it is a material at the mesoscopic scale, rice husk (RH) finds application in composite materials as a reinforcing filler.^[40]^ RH is an agricultural byproduct with a production amounting to ≥120 million tons per year, of which only around 20 million tons are currently used. This leaves 100 million tons unexploited, which can be used for valorization in the context of the circular economy. Its physical characteristics are interesting, because it is a low density, yet highly robust composite material, formed by about 20 % SiO_2_, about 5 % of inorganic oxides and 70–80 % organic components, the majority of which are cellulose, hemicellulose and lignin.^[41]^ Milled RH, that was previously used for enzymes immobilization,^[42]^ is the material with a good granulometry for envisioning its application as polymer filler.

In order to simplify the composition of RH in our functionalization experiments and in the following characterizations, the milled RH particles, sieved to a size of 200 to 400 μm, were delignified by treatment with NaOH and H_2_O_2_. The protocol was developed by modifying and then optimizing methods previously reported in the literature, for the removal of lignin and hemicellulose.[^43^, ^44^] During this process, hydrogen peroxide is spontaneously decomposed according to the Scheme S1, yielding hydroperoxide anion HOO^−^, unable to directly depolymerize lignin. Instead, the resulting hydroxyl radical ⋅OH, is a powerful oxidant responsible for lignin and hemicellulose depolymerisation. The formation of the radical species follows a first order kinetic and is favoured by an alkaline pH of 11.5.[^45^, ^46^] Cellulose shows recalcitrance to degradation by the aqueous protocol applied for delignification, contributing to the retention of the three‐dimensional structure of delignified rice husk (d‐RH). This feature is demonstrated by the stereoscopic microscopy images acquired on RH and d‐RH, shown in the Figure S8, which witnesses the change in colour of the sample accompanying delignification, and by the FE‐SEM micrographies before and after the delignification treatment, shown in the Figure 6a–b, which demonstrate the retained rice husk 3D structure after delignification. Furthermore, the oxidation broke the diferulic linkages between cellulose and lignin, contributing to expose a higher fraction of cellulose of RH to the following functionalization with ELA.

Details of the characterization of RH and d‐RH are collected in the Table 2. Lignin removal increased the ash content in d‐RH with respect to RH. In detail, we quantified ashes in RH and d‐RH, finding 11.68 w/w % and 14.57 w/w % of ashes, respectively, which can be mainly ascribed to the presence of silica. This is corroborated by a higher humidity content in d‐RH than in RH (14.2 % vs 9.6 %). Conversely, values for the water adsorption capacity return similar after soaking in water, with d‐RH retaining 43.6 % vs 42.6 for RH. Delignification also decreases the bulk density of milled RH, whilst, from a porosimetric point of view, the two samples are equivalent, with a slight increase in microporosity of d‐RH (showing 0.0966 μm as average pore diameter vs 0.0888 μm found for RH). From the measured values, it is evident that delignified rice husk is much more prone to wetting than non‐delignified rice husk. This is consistent with the fact that lignin, the most hydrophobic component of rice husk, has been removed from the material during the delignification process.

**Table 2:** Characterization data of rice husk (RH) and delignified rice husk (d‐RH).

Sample	Ash [w/w %]	CRI from XRD^a^	Density [g/mL]	Water retention [%] (retention after soaking)	Total Intrusion Volume [mL/g]	Total pore area [m^2^/g]	Average pore diameter [μm]	Porosity [%]
RH	11.68	71.7 %	0.437	9.6 (42.6)	0.4162	18.740	0.0888	41.3
d‐RH	14.57	66.0 %	0.354	14.2 (43.6)	0.3811	15.783	0.0966	39.3

^a^ Crystallinity index estimated by the Segal method from X‐ray diffraction (XRD) spectra, according to.^[47]^ XRD spectra are reported in the Supplementary Data, Figure S10.

Finally, we could evaluate crystallinity indexes (CRI) of cellulose for RH and d‐RH by the Segal method from X‐ray diffraction (XRD) spectra.^[47]^ The relevant spectra are reported in the Supplementary Data in Figure S9. We observe a CRI decrease after delignification (from 71.7 % to 66.0 %), sign of a slight cellulose hydrolysis during the delignification treatment. XRD spectra demonstrate the presence of cellulose I_β_ and amorphous phase in the two materials with a wide signal centred at 2Θ angle of 16.0, corresponding to coalesced (1–10) and (110), and two peaks centred at 2Θ angles of 22.1 and 34.7, which could be assigned to (200) and (004), respectively.[^31^, ^48^]

**Figure 4:**
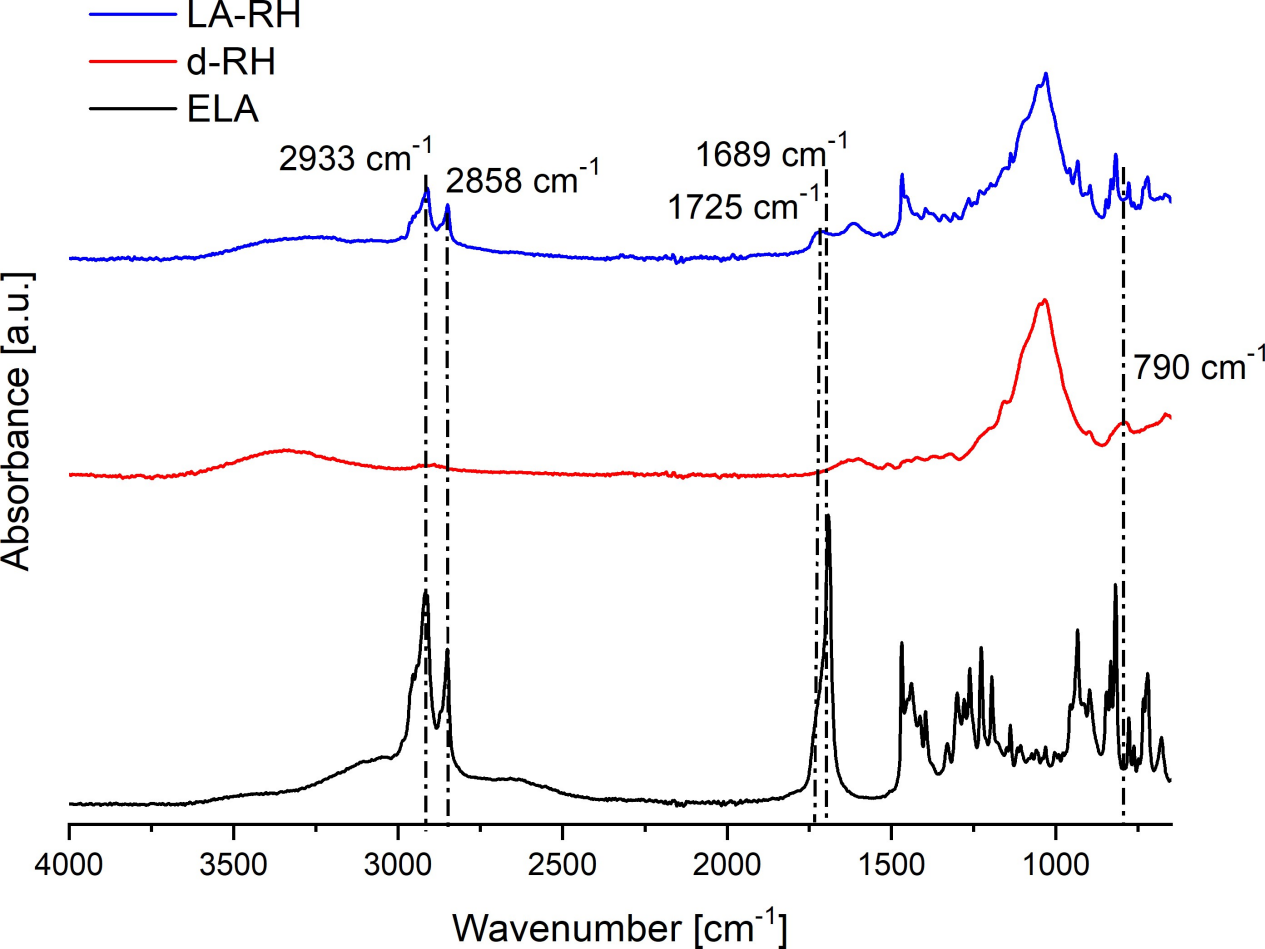
ATR‐FTIR spectra of epoxidized linoleic acid (black line), delignified rice husk (red line), functionalized rice husk LA‐RH (blue line).

After isolation of d‐RH, enriched in cellulose and silica, the IRTA surface functionalization with ELA was carried out under solvent‐free conditions as described on the cellulose nanocrystals. *p*TSA was used as catalyst as in the previous case. The ATR‐FTIR spectra of the delignified RH, ELA and the reaction product, named LA‐RH, are shown in the Figure 4. Delignified rice husk (red line) shows the typical profile of cellulose, with characteristic absorption peaks of acetal units (~1000 cm^−1^). Silica in d‐RH produces a peak centered at 790 cm^−1^ assigned to the symmetric vibrations of Si‐O‐Si bonds. The broad peak at ~1100 cm^−1^ is overlapped to the acetal bonds stretching,^[49]^ and corresponds to antisymmetric vibrations of Si‐O‐Si bonds. Lignin has been apparently completely removed for the lack of diagnostic signals (1610 cm^−1^, and signals of C=C double bonds). Figure S10 shows more in detail the decrease in intensity of the absorption peaks corresponding to lignin (between 1600 and 1650 cm^−1^) and hemicellulose (1730 cm^−1^), removed by the delignification process. This is accompanied by an increase in the intensity of a diagnostic peak attributed to silica (865 cm^−1^, Figure S11). After the functionalization reaction, ATR‐FTIR spectra showed the presence in the functionalized compound LA‐RH (Figure 4, blue trace) of specific signals at 1689 cm^−1^ and the couple of signals around 2880 cm^−1^, related to linoleic acid. Furthermore, we also see in LA‐RH the appearance of a stretching related to ester formation at 1725 cm^−1^. The peak at 790 cm^−1^, corresponding to silica, is covered by signals produced by the linoleic acid molecular skeleton.

**Figure 5:**
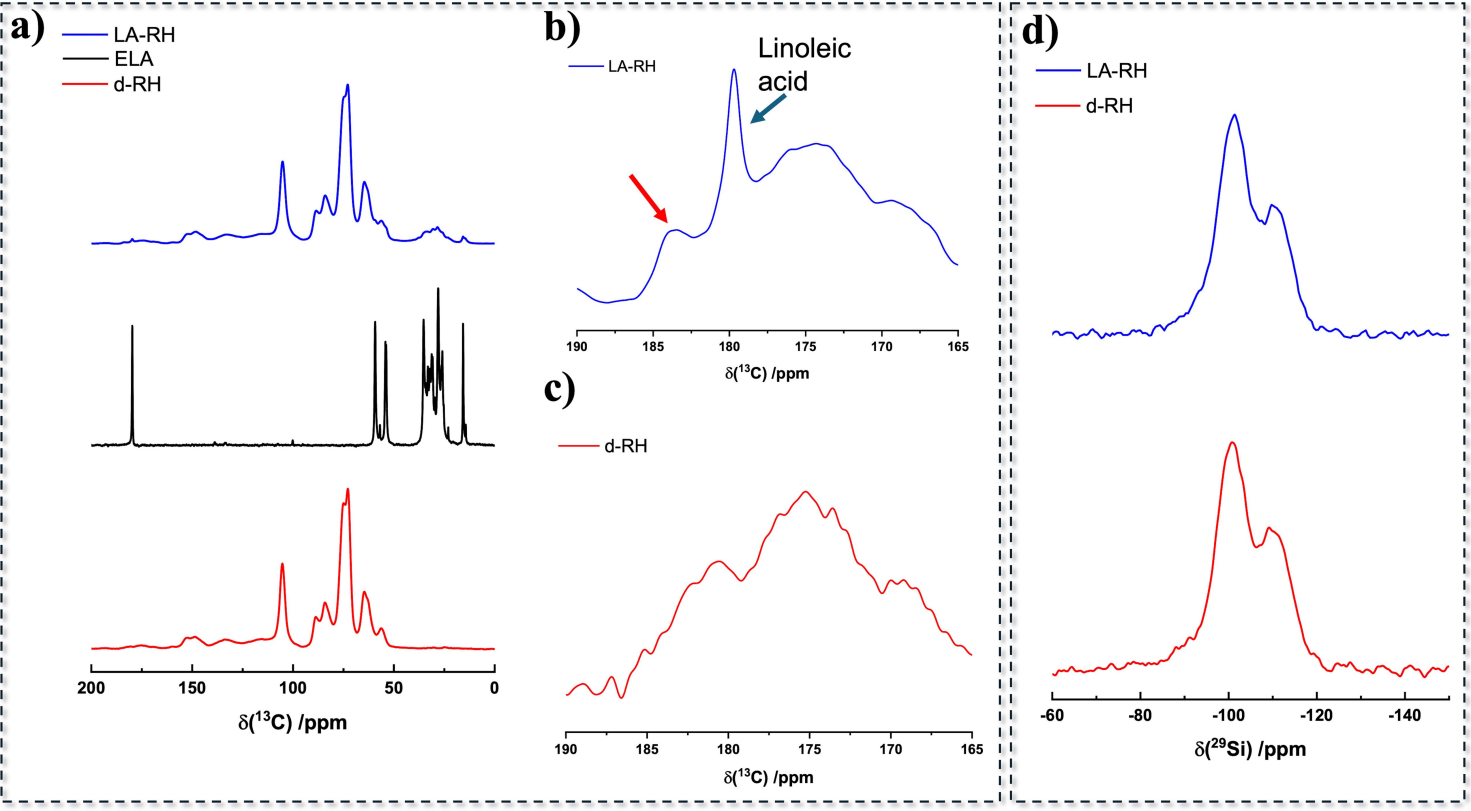
a) ^13^C CP MAS of d‐RH (red trace), ELA (black trace) and LA‐RH (blue trace); magnification of ^13^C CP MAS of LA‐RH (a) and d‐RH (b) in the ester region; d) ^29^Si CP MAS NMR spectra of d‐RH (red trace), and LA‐RH (blue trace).

Following, solid state NMR spectra were acquired for delignified RH and for LA‐RH. The ^13^C CP MAS NMR spectra are shown in the Figure 5a. The delignified RH shows the typical signals of cellulose, plus some residual absorption signals attributable to lignin′s aromatic structure (chemical shift between 150 and 130 ppm).^[50]^ The spectrum of LA‐RH shows the appearance of signals attributed to the linoleic acid functionalization, with aliphatic carbons between 15 and 40 ppm and the appearance of the carboxyl signal of linoleic acid at 180 ppm (Figure 5b). The enlargement of the relevant region of the spectrum shows more intense absorption along with the appearance of a new signal (red arrow) with respect to the spectrum of d‐RH centered at ~185 ppm.

Furthermore, considering the presence of biogenic silica in rice husk, that could potentially be modified during the functionalization reaction, we also acquired solid state ^29^Si CP MAS NMR spectra of the samples to furnish information about the chemical/structural environment of silicon atoms in the biogenic SiO_2_ matrix. First, we conducted an analysis on the starting RH and d‐RH. The two relevant spectra are shown in the Supplementary Data, Figure S12b. The ^29^Si CP MAS NMR spectra present two main peaks at −100 and −112 ppm, ascribed to Q3, [Si(Si‐O)_3_(OH)], and Q4, [Si(Si‐O)_4_].^[51]^ The pristine RH displayed a further signal falling at −93 ppm, attributable to silicon atoms in silane diols groups Q2, [(OH)_2_⋅Si(OSi)_2_], which decreases in intensity with the delignification. Conversely, the spectrum of biogenic silica does not appear modified after the IR triggered reaction with ELA (Figure 5b), pointing at the negligible influence of the reaction on the chemical environment of silanol units

Elemental analyses on d‐RH and LA‐RH revealed an increase in carbon content from 37.47 % to 41.57 % after the functionalization with epoxidated linoleic acid (Table 3). In this case, we could not calculate a degree of substitution, due to the complex composition of d‐RH. However, by attributing the overall sulfur increase to *p*‐toluensulfonate and the remaining carbon percentage increase to linoleic acid, we derive absorption of 0.017 mol of ELA (corresponding to 5.25 g of ELA) per 100 g of d‐RH.

**Table 3:** Elemental analyses of d‐RH and LA‐RH.

Sample	Elemental Analyses				
	C	H	N	S	O^a^
d‐RH	37.47	4.81	0.13	0.02	43.00
LA‐RH	41.57	5.79	0.44	0.18	52.02

^a^ Oxygen content determined by difference after ash correction.

**Figure 6:**
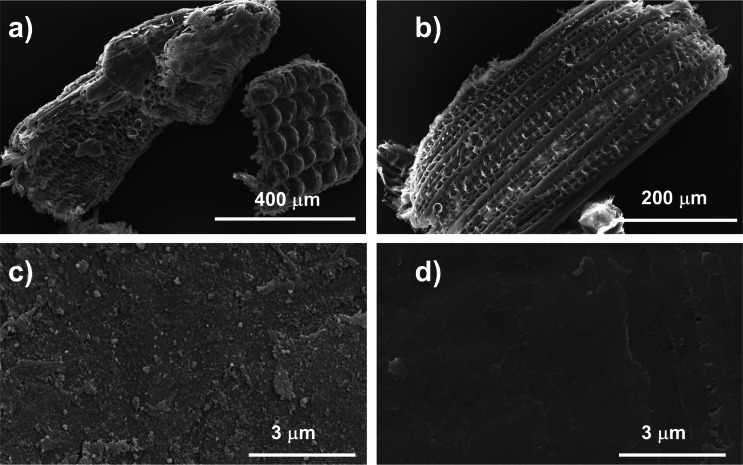
FE‐SEM micrographs of a) RH after milling and sieving, with granulometry between 200–400 μm; b) d‐RH prepared from milled and sieved RH; c) d‐RH surface at higher magnification; d) d‐RH surface after reaction with ELA (LA‐RH).

The changes in RH morphology, occurring during delignification and during the following reaction with ELA, were assessed acquiring FE‐SEM micrographs. The Figure 6a shows the morphology of RH used in this study. As deducible from the data of Table 2, the delignification applied to RH was respectful of the 3D morphology of RH. Delignified RH, reported in Figure 6b, conserves, indeed, all the features of the starting material. For detecting the morphology changes due to the functionalization reaction, we observed a detail of rice husk′s honeycomb structure, in particular one of the hollow alveoli. The micrographs are shown in the Figure 6c‐d. The comparison of the same kind of detail in delignified rice husk (Figure 6c) and the same sample after the reaction (Figure 6d) clearly demonstrates an effective coverage of rice husk′s surface by organic material.

**Figure 7:**
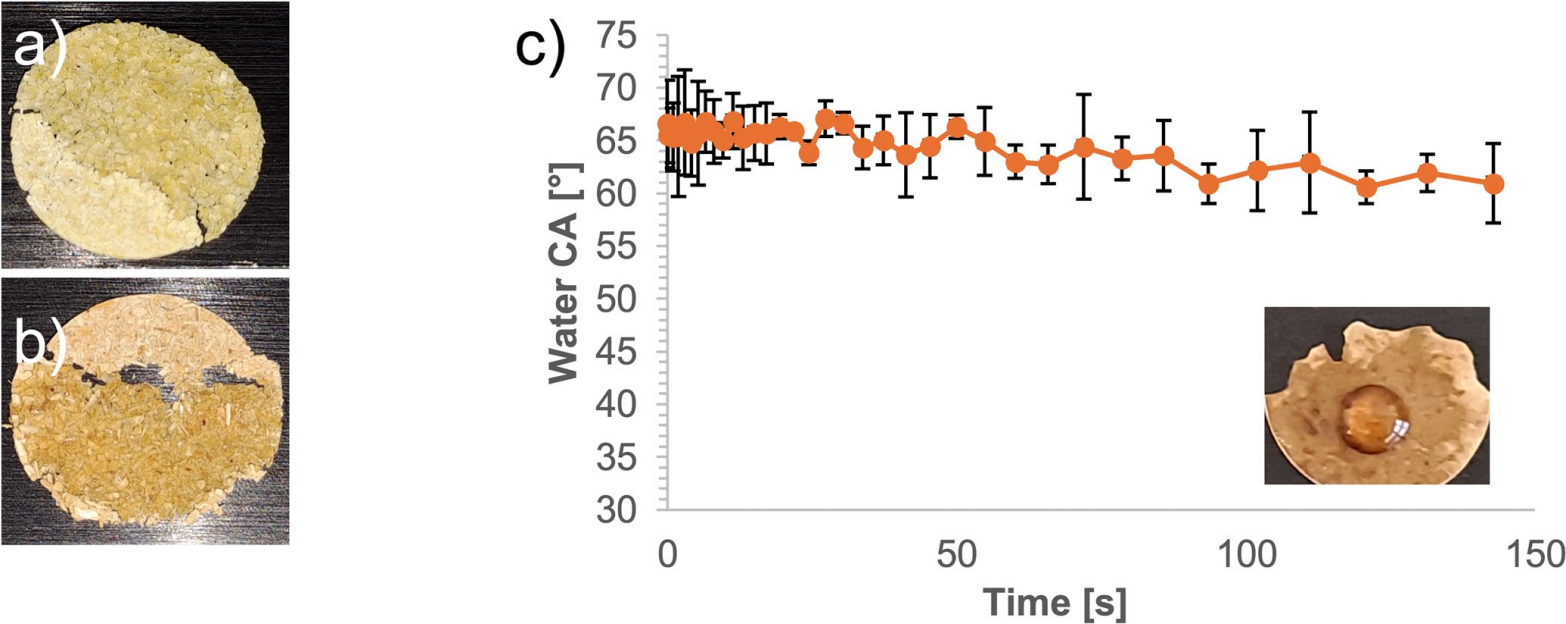
Pressed samples of d‐RH (a) and RH (b) after water contact angle measurement attempts. It is evident the wet area and the sample disaggregation. c) Contact Angle evolution with time of water on the surface of a pressed LA‐RH sample. Digital photo of the water droplet deposited on LA‐RH in the inset.

Water static contact angle measurement was performed on RH, d‐RH and LA‐RH to understand the effects of the treatment applied to rice husk on its surface chemistry. In the case of RH, for its mesoscopic dimension and poor dispersibility in any solvent, the samples could not be drop‐cast on glass from DMSO suspensions as cellulose nanocrystals. Instead, the water contact angle measurements were carried out on pressed samples. The first macroscopic difference was given by the difficulties of sample preparation and analysis encountered for RH and d‐RH. Pressed samples were irregular and extremely brittle. A water contact angle measurement was attempted, but the water drop penetrated instantaneously into the pressed samples, leading also to their disintegration (Figures 7a,b and S14). This behavior was expected by the observed water retention abilities of the two samples (Table 2). After the reaction with ELA, the initial water contact angle on the surface of the LA‐RH sample was equal to 65°. The value decreased to 60° after ~100 s from drop deposition and then remained constant (Figure 7c). The measurements showed repeatability as observed for the cross‐linked nanocellulose films (compare Figure S13). Hence, the successful functionalization of d‐RH was further clearly demonstrated by the macroscopic changes in the water contact angle and, therefore, in sample wettability.

## 3 Conclusions

In this study, we have illustrated for the first time a solvent‐free infrared thermal assisted (IRTA) approach for the surface functionalization of cellulose‐based nano‐ and meso‐materials. By coupling cellulose with epoxidated linoleic acid (ELA) in the presence of *p*TSA as catalyst, we are able to functionalize cellulose nanocrystals achieving a degree of substitution of 0.09, which is high enough to reveal a modification in the surface energy of nanocrystals thin films, towards their hydrophobization. We achieve an in deep characterization of the materials at the molecular level, by applying several characterization techniques which include ATR‐FTIR, liquid and solid‐state NMR, elemental analyses and water contact angle. Using the CNCs as reaction models composed of pure cellulose, we then extend the reaction procedure to milled and delignified rice husk. The material used in this work, with its structural recalcitrance to degradation, appears the ideal substrate to test the validity of the proposed functionalization approach. By applying the same characterization techniques, we demonstrate the effectiveness of the modification reaction also applied to d‐RH. In addition, whilst the functionalized LA‐RH can be processed into films by pressing technique, the starting materials RH and d‐RH show inefficiency in yielding stable pressed layers. Our functionalization enables, therefore, new applications for LA‐RH. The hydrophobized RH is a cost‐effective material, potentially valuable in several technological fields, with the advantage of being generated, according to our synthetic approach, with minimized energy expenditures and use of organic solvents, employing reactants deriving from enzymatic synthesis (ELA), and complying with the main principles of green chemistry.

## Conflict of Interests

The authors declare no conflict of interest.

4

## Supporting information

As a service to our authors and readers, this journal provides supporting information supplied by the authors. Such materials are peer reviewed and may be re‐organized for online delivery, but are not copy‐edited or typeset. Technical support issues arising from supporting information (other than missing files) should be addressed to the authors.

Supporting Information

## Data Availability

The data that support the findings of this study are available from the corresponding author upon reasonable request.
