# Supplementary material for: Cellulose Nanocrystals and Rice Husk Surface Functionalization Induced by Infrared Thermal Activation
Source: ChemSusChem. 2025 Mar 27;18(11):e202500164. doi: 10.1002/cssc.202500164 (PMC12131702; doi:10.1002/cssc.202500164)
Supplement: Supplementary file 1 — Supporting Information [file CSSC-18-e202500164-s001.pdf]

ChemSusChem

Supporting Information

# Cellulose Nanocrystals and Rice Husk Surface Functionalization Induced by Infrared Thermal Activation

Rosarita D'Orsi, Chiara Danielli, Mariachiara Spennato, Elisa Guazzelli, Elisa Martinelli, Fioretta Asaro, Lucia Gardossi,* and Alessandra Operamolla*

Supporting Information

# Cellulose Nanocrystals and Rice Husk Functionalization Induced by Infrared Thermal Activation

Rosarita D'Orsi[1,2], Chiara Danielli[3], Mariachiara Spennato[3], Elisa Guazzelli[1], Elisa Martinelli[1], Fioretta Asaro[3], Lucia Gardossi,[3,*] and Alessandra Operamolla[1,2,*]

[1] Dipartimento di Chimica e Chimica Industriale, Università di Pisa, via Giuseppe Moruzzi 13, 56124, Pisa, Italy

[2] Consorzio Interuniversitario Nazionale di ricerca in Metodologie e Processi Innovativi di Sintesi C.I.N.M.P.I.S. 56124 Pisa, Italy

[3] Department of Chemical and Pharmaceutical Sciences, University of Trieste, Via L. Giorgieri 1, 34127 Trieste, Italy

Corresponding authors:
Lucia Gardossi gardossi@units.it
Alessandra Operamolla alessandra.operamolla@unipi.it

**General Remarks**

All reagents were purchased at the highest commercial quality and used without further purification. Lipase B from *Candida antarctica* (CaLB Novozyme 435), commercialized by Novozymes (Denmark), displayed a specific activity of 1998 U/gdry according to the tributyrin hydrolysis assay. Avicel PH-101 was used as the starting material for nanocrystalline cellulose isolation. Avicel has a declared molecular weight of 36 kDa with a degree of polymerization of 220. However, we characterized previously Avicel and we found a polymerization degree of 153 with a dispersity of 1.35. [15, 22] Avicel contains 95.4% of cellulose, 2% humidity, 2% of hemicellulose (in the form of xylan) and 0.6% of lignin. Sonication of nanocellulose suspensions was carried out with a Dr. Hielscher 400 W tip sonicator. Dialysis was carried out at room temperature against deionized water in nitrocellulose tubes with a cut off of 12600 Da. S_CNCs and N_CNCs were synthesized as described elsewhere. [15, 22] Rice husk was kindly donated by Riseria Cusaro, Binasco (MI, Italy), milled with a blade mill (VERDER SCIENTIFIC S.r.l., Bergamo) and sieved to obtain a fraction with dimension 200-400 μm as previously reported [42]. The IR lamp used for the thermal activation was a Philips Infrared Industrial Heat Incandescent lamp Br125 IR 250W 230–250 VCL 1CT. The IR lamp and experimental design is the same used by others in reference [16]. The IR lamp was characterized, in terms of emission spectrum and irradiance, by Mazzotta et al. [53] The temperature at the reaction stage was monitored with a thermocouple. For the evaluation of reaction yields on cellulose nanocrystals, weight quantification was done considering the weight of isolated product, with respect to the total weight of the reagents. For the evaluation of reaction yields on rice husk, the total weight of the isolated material was divided by the total weight of the two reactants.

**Solventless Chemo-Enzymatic Epoxidation of Linoleic Acid**

The epoxidated linoleic acid (ELA) was synthesized according to [26]. The reaction was performed on a 2 g scale in the presence of CaLB (450 U per g of substrate). The reagents were mixed in a 25 mL round flask for 15 min at 50°C. After this time, $H_2O_2$ (2 equivalents with respect to linoleic acid) was slowly added to the reaction system. After 3 h total reaction time, the product was extracted with dichloromethane (3x20 mL). The organic phase was collected, dried over anhydrous sodium sulfate, and the dichloromethane was distilled under reduced pressure at temperature below 40°C. The conversion of C=C into epoxy functionalities was evaluated by $^1$H NMR. The product was also analyzed by GC-MS.

## Reaction between sorbitol and ELA

### Thermal heating

The ring-opening reaction was carried out in a nitrogen atmosphere in a 25 mL Schlenk tube equipped with a magnetic stirrer and a vertical bubble condenser. Epoxidized linoleic acid (ELA, 0.052 g, 0.16 mmol) was heated at 120° C and then sorbitol (0.044g, 0.24 mmol) was added under a nitrogen flux. After the mixture had melted, 2 ml of sulfuric acid was added as catalyst. After three hours at 120°C, the product with an intense yellow colour was quenched with aqueous ammonia at 30% by weight, and the mixture was centrifuged to recover the solid. The compound was dried in an oven at 40°C for one night and characterized. For comparison a test was performed with 50 mg of epoxidized linoleic acid and sulfuric acid at 120°C. NMR spectra in $CDCl_3$ showed that epoxydic signals disappeared with acidic conditions.

### IR thermal activation

The reaction was carried out in air in a 25 mL beaker equipped with a magnetic stirrer under IR lamp irradiation. The temperature of the hotplate was monitored with a thermocouple and the maximum temperature reached was 120° C. Epoxidized linoleic acid (0.094 g, 0.30 mmol) was melted by IR irradiation. After 5 minutes, the sorbitol (0.080 g, 0.46 mmol) was added. After the reagents were dissolved, 2 ml of sulfuric acid or 8 mg of *p*-toluensulfonic acid (5% w/w with respect to the mixture) was added as catalyst and the reaction was carried out for 15 minutes. At the end of the reaction, the product was quenched with aqueous ammonia at 30% by weight (3 mL) and deionized water (30 mL), and the mixture was suspended in dichloromethane. The crude was extracted with dichloromethane (3x30 mL). The organic phase was collected and washed with brine (3x30 mL) followed by deionized water (3x30 mL). The organic phase was dried over anhydrous sodium sulfate, filtered and the solvent was distilled under reduced pressure. A white solid was isolated (81 mg).

**Rice Husk Delignification**

Delignification was carried out according to the Scheme S1. 50 g of milled rice husk (particle size 200-400 μm) were introduced in a 5 L beaker and suspended in 200 mL of $H_2O_2$ (20% v/v); the mixture was stirred for 10 minutes to fully soak the rice husk, and heated up to 70°C, introducing the temperature measuring probe directly in the reaction mixture. Then, 1 g of NaOH was added to the mixture; the formation of a great volume of foam is observed. Subsequent 1 g aliquots of NaOH were added to the mixture after 45 and 90 minutes from the first; each time, 150 mL of $H_2O_2$ were also added to the mixture. The mixture was kept under magnetic stirring, at 70°C, for a total time of 5 hours starting from the first NaOH addition. The resulting material is light yellow in color. The delignified rice husk was then filtered under reduced pressure and washed with 6 × 50 mL of $H_2O$, until complete neutralization of the wash water. The final product was dried in the oven at 100°C for 2 hours.

$$H_2O_2 = H^+ + HOO^-$$
$$H_2O_2 + HOO^- = \cdot OH + O_2^- \cdot + H_2O$$

**Scheme S1.** Hydrogen peroxide decomposition in alkaline conditions

**Characterization of delignified rice husk**

The rice husk was characterized with a stereomicroscope (Leica MZ 16, Leica Microsystems, Wetzlar, Germany), equipped with a Nikon E4500 camera (Nikon, Japan). The porosity of the material was determined with a mercury porosimeter. Before the density measures, the rice husk (delignified, particle size 200-400 μm) was dried in the vacuum oven (80°C, overnight) in order to remove water adsorbed by the matrix. For the density measurements, a 100 mL graduated cylinder was filled with anhydrous rice husk up to the 100 mL mark. The material was pressed to make it even and compact. The rice husk was then weighed, and the bulk density was determined by:

$$density\ (g\ mL^{-1}) = \frac{weight_{rice\ husk}\ (g)}{100\ mL}$$

Water retention capacity of RH was measured by an established procedure, [25] using a series of soaking treatments with increasing $H_2O$/EtOH ratios to promote the full wettability of the matrix and the retention of water in the tunnels of the material. This treatment is necessary as raw rice husk has a high content in lignin (31% of the organic components of

rice husk). The results were compared with control samples of RH that did not undergo the soaking treatment.

**Synthesis of N_CNCs**

300 mL of deionized water were introduced in a four necked 1 L cylindrical glass reaction flask equipped with a mechanic glass stirring bar, a nitrogen inlet and an air condenser. Then, the flask was cooled in an ice bath and 300 mL of concentrated HCl were added. After that, 30 g of Avicel PH-101 were added and the suspension was warmed to 105 °C for 5 h. The system was cooled to room temperature and left to rest overnight under stirring. Then, it was diluted with 200 mL of distilled water and the mixture was transferred to polypropylene centrifugation tubes. Centrifugation at 4000 rpm for 10 min was repeated 4 times replacing the supernatant solution with fresh deionized water until the pH was approximately 3. The precipitate was dialyzed against distilled water until neutrality using a cellulose nitrate membrane with a molecular weight cut-off of 12,400 Da. The resulting suspension was sonicated with a tip sonicator, centrifuged at 1000 rpm for 10 minutes. The supernatant was recovered, and water was removed by freeze drying, yielding cellulose nanocrystals. The lateral and longitudinal dimensions of CNCs were evaluated from FE-SEM micrographies with 120000 magnifications, using the software ImageJ 1.53e, Institute of Health, USA on the average of >50 measurements. N_CNCs presented average length 151±18 nm and lateral dimension 10±2 nm. N_CNCs did not show net surface charge, since the hydrolysis reaction does not introduce any functionality on the surface other than the native hydroxyl groups of cellulose. Furthermore, attempts of acquisition of the zeta potential in water suspension was unsuccessful due to sample fast aggregation. [15, 22]

**Cross-linking of N_CNCs with ELA**

N_CNCs (0.400 g, ~2.5 mmol of cellulose) and epoxidated linoleic acid (ELA, 0.298 g, ~1 mmol) were grounded using a mortar and a pestle. Then, *p*-toluenesulfonic acid (5% w/w) was added as catalyst and grounded in the same mortar. The mixture was stirred in a beaker under IR illumination until ELA melted and the mixture became homogeneous. The reaction was conducted for 15 minutes after melting of LA. At the end of the reaction, the product, with an intense yellow color, was cooled and quenched with a solution of aqueous ammonia at 30% by weight and was recovered by centrifugation. Cross-linked nanocrystals were dried in the oven at 40°C for one night and 0.430 g of yellow powder was obtained and characterized (~62% mass yield).

**Reaction of delignified rice husk with ELA**

Delignified rice husk with a granulometry 200-400 μm (0.206 g) and epoxidized linoleic acid (0.158 g ~0.5 mmol) were grounded using a mortar and a pestle. Then, *p*-toluenesulfonic acid (0.004 g 2% w/w) was added as catalyst and grounded in the same mortar. The mixture was stirred in a beaker under IR irradiation until the linoleic acid melted and the mixture became homogeneous. After 15 minutes, the product was cooled and quenched with a solution of aqueous ammonia at 30% by weight, and then it was recovered by centrifugation. The product was dried in the oven at 40°C for one night and 0.220 g of yellow powder was obtained and characterized (61% of yield).

**ATR-FTIR analysis**

ATR-FTIR spectra on CNCs and their reaction products were collected with a Thermo Fischer Nicolet iS50 FTIR instrument interfaced with an ATR ITX accessory equipped with a diamond crystal (radiation penetration approximately 2 μm at 1000 $cm^{-1}$). The spectra were recorded at room temperature in air in the range between 4000 and 650 $cm^{-1}$ with a resolution of 4 $cm^{-1}$, 16 accumulated scans, and a DTGS as a detector. The spectra were elaborated using SPECTRA software.

**Elemental analysis**

Elemental analyses were performed on an Elementar Vario Micro Cube analyser. Oxygen content was calculated for all samples by difference. All determinations were done in duplicate. The standard deviation was always lower than 0.2.

The ash content was determined using a muffle furnace (model Hulk MSW-Z51) at the temperature of 525 °C for 16 h. All the determinations were done in duplicate on 500 mg samples in porcelain crucibles.

**$^{1}H$ NMR Spectroscopy**

Experiments of cross-linked sorbitol were performed as follows: 15 mg of sample were dissolved in 0.8 mL of deuterated chloroform (ELA and reaction product) or DMSO-$d_6$ (sorbitol). NMR measurements were carried out on a JEOL YH spectrometer with a probe operating at 500 MHz. The spectra were analysed using JEOL Delta software.

To perform $^{1}H$ NMR experiments on cellulose nanocrystals, 15 mg of samples were suspended in 0.7 mL of DMSO-d6 (99.96% D) using a tip sonicator (Duty Cycle constant, Power 60 W for 60 seconds). Proton spectra were obtained using a JEOL YH spectrometer with a probe operating at 500 MHz and 65°C, according to our previously published procedure. [14] Typical conditions of solvent suppression experiment employed were 5 s relaxation delay, 256 or more scans, wet number 2 and the offset were around 3.11 and 2.46 ppm relative to the $H_2O$ and DMSO resonance respectively. The spectra were analyzed using JEOL Delta software.

**$^{13}C$ CP/MAS solid state NMR spectroscopy**

The $^{1}H$->$^{13}C$ CP/MAS NMR spectra were recorded at the Centre for Instrument Sharing of the University of Pisa (CISUP) using a Bruker Avance NEO 500 equipped for high-resolution studies on solid systems, with a CP/MAS probe hosting rotors with an outer diameter of 4

mm. The spectrometer operates at a static magnetic field of 11.7 T, corresponding to a Larmor frequency of 125 MHz for 13C nuclei. The MAS frequency was 10 kHz. More than 2000 experiments were acquired to provide a total acquisition time ≥2 h. The CP/MAS $^{13}C$ NMR method generates signals from carbon atoms of the glucopyranose unit of cellulose. Anomeric C1 gives a peak in the range of chemical shift (δ) of 100–110 ppm; C2, C3, C5 in the range of 70–80 ppm; and C6 in the range of 60–68 ppm. [48] In the range of 82–90 ppm we can find the peaks of C4, in particular the peak at 88–89 ppm is attributed to the C4 atoms of the crystallites, while the peak at 83-84 ppm is attributed to the C4 atoms of non-crystalline domains. The “crystalline” and “non-crystalline” peaks of C4 atoms are used to estimate the crystallinity index, after a Voight deconvolution procedure. The crystallinity index was calculated according to the following equation:

$$CI\ (\%) = 100 \cdot \frac{F_{cr}}{(F_{cr} + F_{nc})}$$

where $F_{cr}$ and $F_{nc}$ are areas of peaks (signals) attributed to C4-atoms of crystalline and non-crystalline domains, respectively.

**FE-SEM**

The Field Emission - Scanning Electron Microscopy (FE-SEM) investigation on rice husk was performed with a Leica Stereoscan 430i scanning electron microscope (Leica Cambridge Ltd., Cambridge, UK). Samples were metallized using a S150A Sputter Coater instrument (Edwards High Vacuum, Crawley, West Sussex, UK).

Field Emission Scanning Electron Microscopy investigation on cellulose nanocrystals, cross-linked cellulose nanocrystals and derivatised rice husk, was performed at the Center for Instrument Sharing of the University of Pisa (CISUP) using a FEI FEG-Quanta 450 instrument (Field Electron and Ion Company, Hillsboro, OR, USA). The cellulose nanocrystals were deposited on glass from DMSO suspension, at a concentration of 1 mg $L^{-1}$. The samples were sputtered with platinum before analysis.

**XRD analysis**

X-ray Diffraction (XRD) analyses were performed at Centro di Servizi di Cristallografia Strutturale of Università degli Studi di Firenze using an X-ray diffraction (XRD) system Bruker D8 Advanced “Da Vinci” operating in the Bragg−Brentano geometry. The diffractometer was equipped with a copper radiation source (Cu Kα-radiation λ = 0.154186 nm operating at 40 kV/40 mA) and a solid-state detector LynxEye. The samples were manually ground with an agate mortar and a pestle before the measurements and deposited

on a zero-background sample holder. The data were recorded with a locked-coupled scan from 5 to 50°, a step size of 0.03°, and 0.5 s per step. The background was subtracted from each diffractogram with the software Bruker DIFFRAC.EVA Version 6. The crystallinity index (CI) of cellulose was calculated from the XRD spectra by the method reported by Segal, [47] according to the following equation:

$$CI\ (\%) = 100 \cdot \frac{I_{200} - I_{am}}{I_{200}}$$

where $I_{200}$ represents the maximum intensity of the peak with Miller′s indexes 200 (centred between 22.4 and 22.6° in cellulose I), while the intensity of the amorphous peak is calculated at the maximum, which depends on the typology of cellulose and is centred at 18° for cellulose I and at 16° for cellulose II.

**Contact Angle Measurement**

Contact angle measurements were conducted using bi-distilled water and *n*-hexadecane. The samples were prepared in the following way. N_CNCs and LA_CNCs samples were dispersed in DMSO at a concentration of 15 mg/mL. Thin films were deposited by solution casting on circular glass slides with 12 mm diameter. For rice husk, delignified rice husk, and rice husk and cellulose nanocrystals functionalised with ELA, samples were prepared by pressing powders into disks using a manual press (Perkin Elmer) and imparting a force of 5 tons for 10 minutes. The samples were analysed with an FTA200 Camtel goniometer.

## $^{1}$H NMR analysis of ELA and cross-linked sorbitol

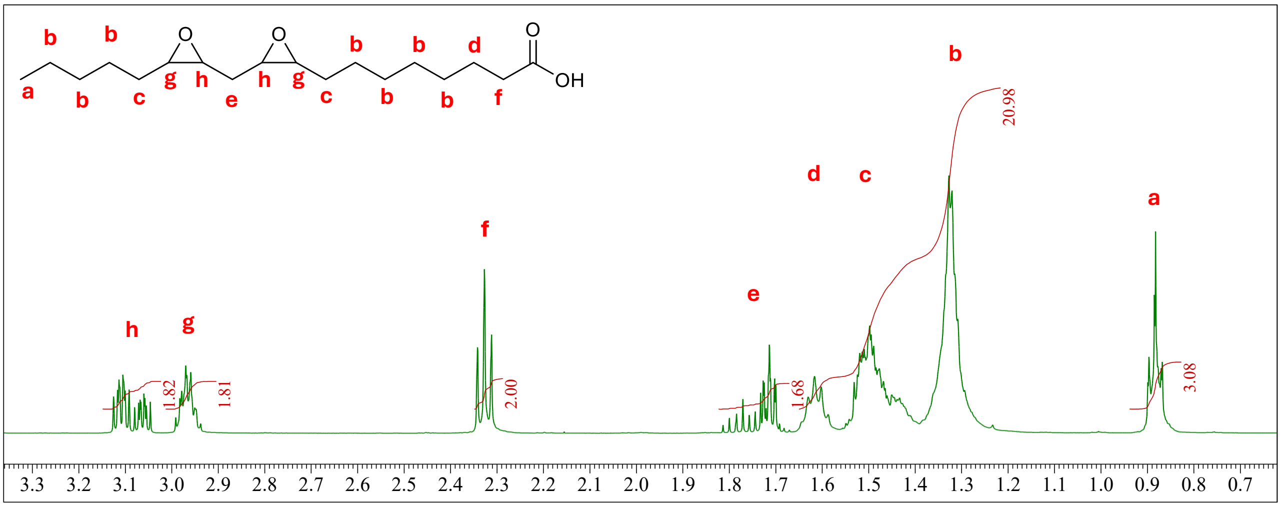


*Figure S1.* [1]*H-NMR spectrum in* $CDCl_3$ *of epoxidized linoleic acid.*

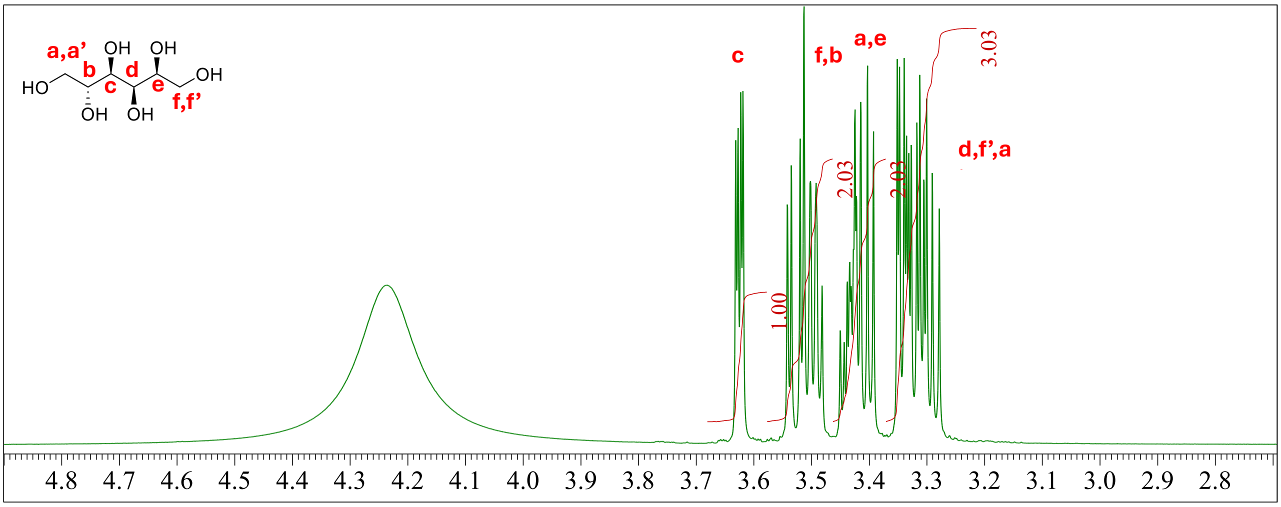


*Figure S2.* [1]*H-NMR spectrum in DMSO-*$d_6$ *of sorbitol.*

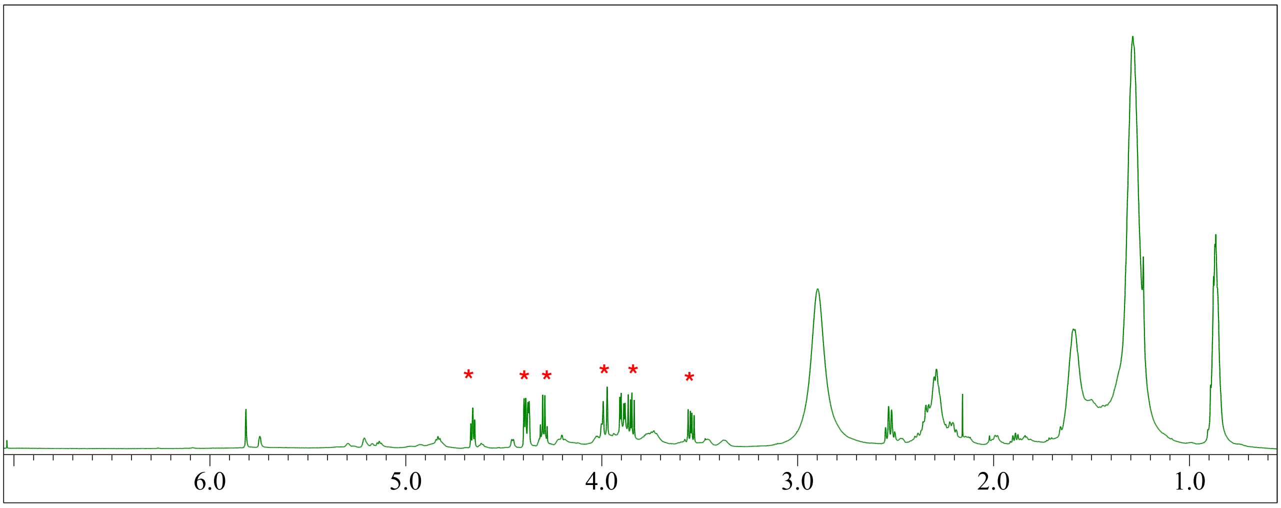


*Figure S3.* [1]*H-NMR spectrum in* $CDCl_3$ *of the product isolated after thermal reaction.*

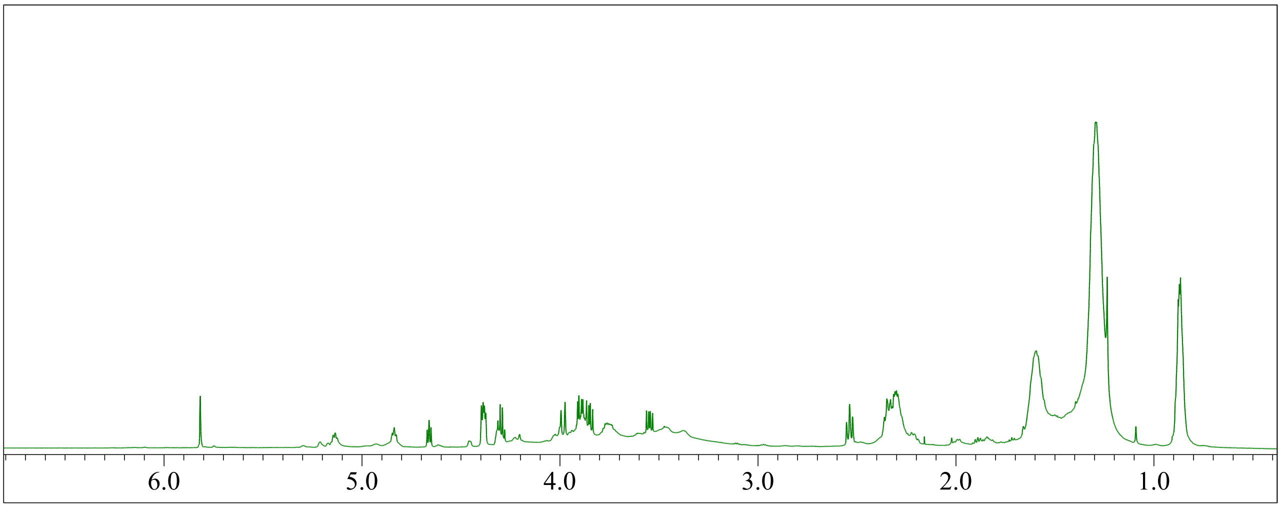


*Figure S4. Enlarged $^{1}$H-NMR spectrum in $CDCl_3$ of cross-linked product isolated after IR thermal activation under H2SO4 catalysis.*

NMR spectra showed that the product isolated after thermal heating (figure S4) and IR thermal activation (figure S5) are similar. In both cases signals referred to linoleic acid molecular skeleton appear broader, whilst the signals of epoxydic protons have disappeared, sign that ring-opening took place. The appearance of sorbitol signals in the products (indicated by a red mark) indicate the presence of the sugar in the polymerized acid. Sorbitol is insoluble in chloroform, and the appearance of signals attributable to it in $CDCl_3$ is a sign that it has been covalently linked to linoleic derivative yielding a chloroform soluble polymer.

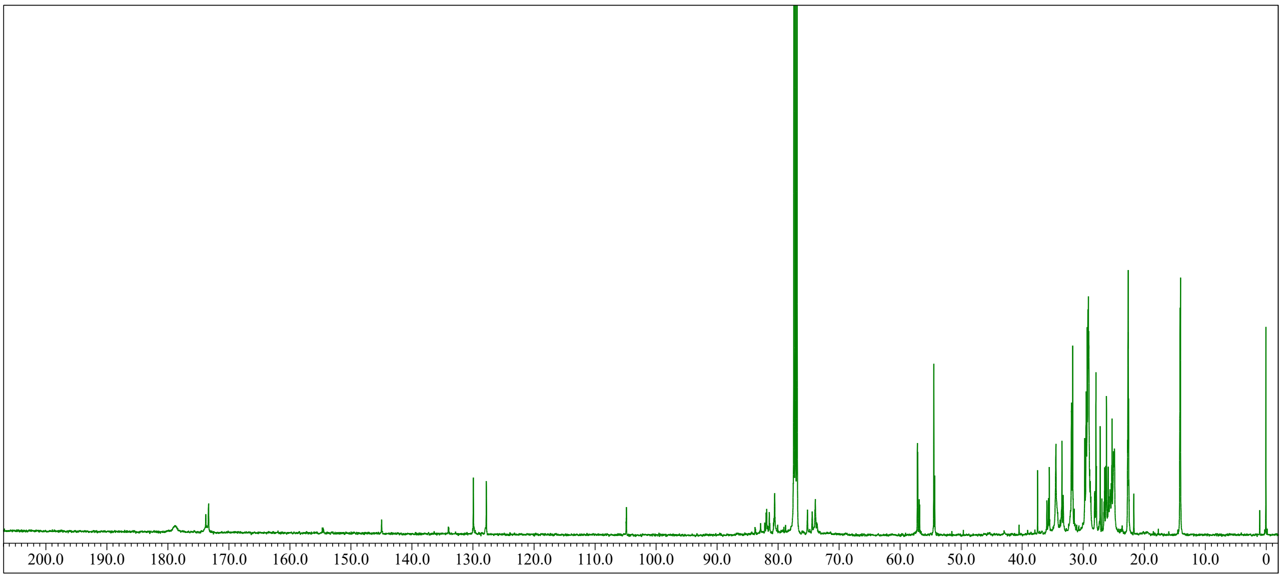


*Figure S5. $^{13}$C-NMR spectrum in $CDCl_3$ of cross-linked product isolated after IR thermal activation under p-toluensulfonic acid catalysis.*

**Approximate calculation of the Degree of Substitution of CNCs from elemental analyses**

The approximate molecular formula for the cross-linked CNCs can be derived from elemental analyses values reported in the Table 1 of the main text. According to them, the composition of the sample is as follows:

$$C_{47.96/12.011}H_{7.12/1.0079}N_{0.29/14.0067}S_{0.76/32.06}O_{43.87/15.9994} \quad (1)$$

$$C_{3.9930}H_{7.0642}N_{0.0207}S_{0.0237}O_{2.7420} \quad (2)$$

The formula (2) has been multiplied times 2 to obtain formula (3):

$$C_{7.986}H_{14.1284}N_{0.0414}S_{0.0474}O_{5.4840} \quad (3)$$

The raw formula for pure cellulose has been grouped in parenthesis, yielding the formula (4):

$$(C_6H_{10}O_5)C_{1.986}H_{4.1284}N_{0.0414}S_{0.0474}O_{0.4840} \quad (4)$$

The nitrogen content was considered as absorbed ammonia, probably as imine or other derivative, giving formula (5):

$$(C_6H_{10}O_5)\cdot(NH_3)_{0.0414}C_{1.986}H_{3.9214}S_{0.0474}O_{0.4840} \quad (5)$$

The sulfur content was completely attributed to *p*-toluensulfonate (-O-$SO_2C_7H_7$), allowing to derive formula (6):

$$(C_6H_{10}O_5)\cdot(NH_3)_{0.0414}\cdot(SO_2C_7H_7)_{0.0474}C_{1.6542}H_{3.5896}O_{0.3892} \quad (6)$$

The content in ELA was considered attributing to each attached ELA unit four hydroxyl groups (-O-$C_{18}O_5H_{35}$), giving formula (7):

$$(C_6H_{10}O_5)\cdot(NH_3)_{0.0414}\cdot(SO_2C_7H_7)_{0.0474}\cdot(C_{18}H_{35}O_5)_{0,0919} \quad (7)$$

The formula (7) respects the found elemental composition, except -0,07 O atoms and +0.3 H atoms per formula.

## Hexadecane Contact Angle

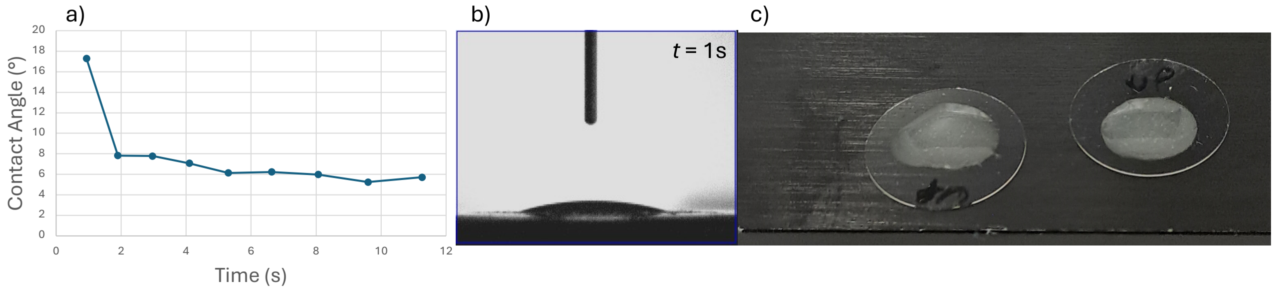


*Figure S6. Normal hexadecane Contact Angle measured over time on LA-CNCs thin films. a) Contact angle evolution in 10 seconds on as deposited thin films; b) photo of the hexadecane drop; c) and d): photos of the drops deposited on the samples.*

## Water Contact Angle

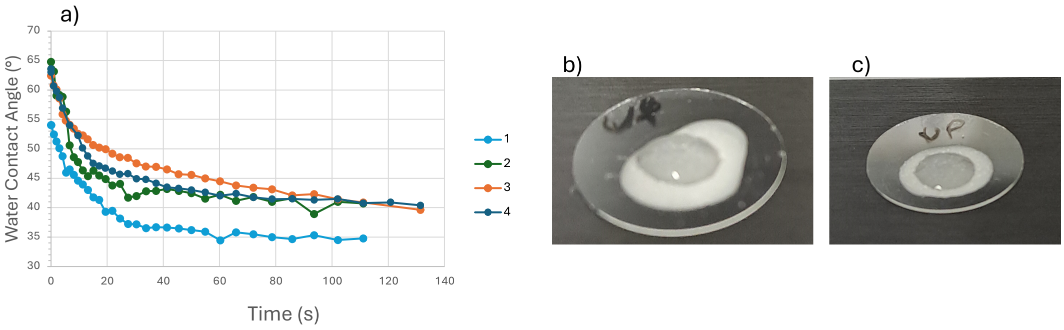


*Figure S7. Static Water Contact Angle measured over time on LA-CNCs thin films. a) Water contact angle evolution in 120 seconds of (1) and (4) as deposited thin films; (2) and (3) repetition of the measurement after drying the film and redepositing a drop. Volume of the drop: 7 uL. Measurement (1) in blue line refers to a drop deposited at the border of the film. b) and c): photos of the drops deposited on the samples.*

## Rice Husk Characterization

A) B)

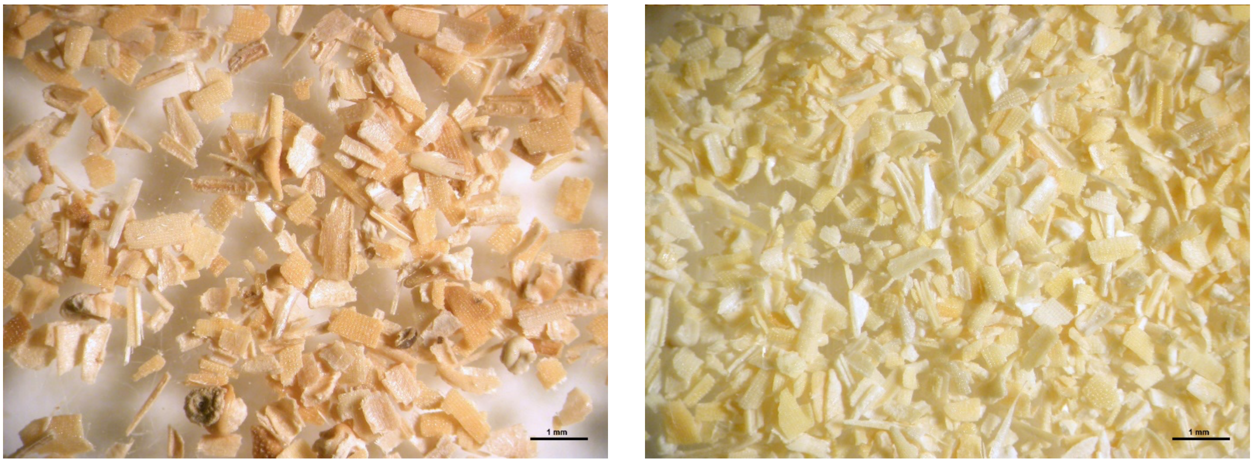

*Figure S8: Stereoscopic microscopy images of raw (A) and delignified (B) milled rice husk, particle size 200-400 µm.*

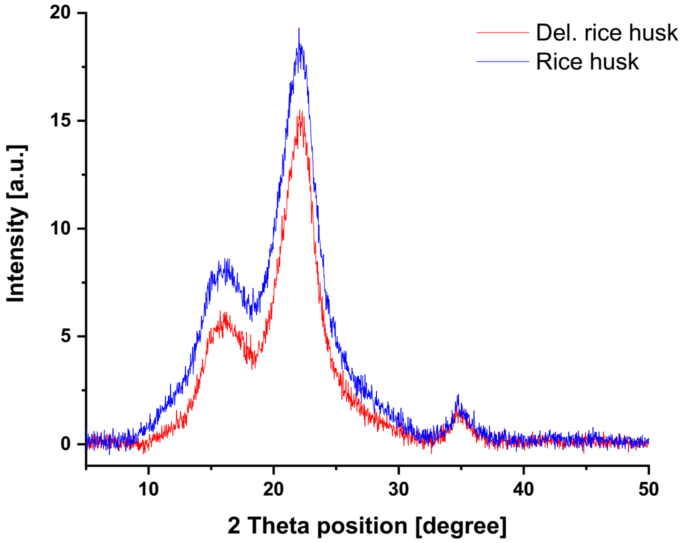


*Figure S9. XRD spectra of delignified rice husk in red line and of pristine rice husk in blue line*

## ATR-FTIR analysis of RH and d-RH

Attenuated Total Reflectance – Fourier Transform Infrared (ATR-FTIR) spectra on rice husk were acquired using a spectrometer FT-IR 4700 JASCO. To acquire the spectrum, the sample was pressed to obtain a homogeneous tablet. The spectrum was acquired in the region between 1800 and 600 cm$^{-1}$.

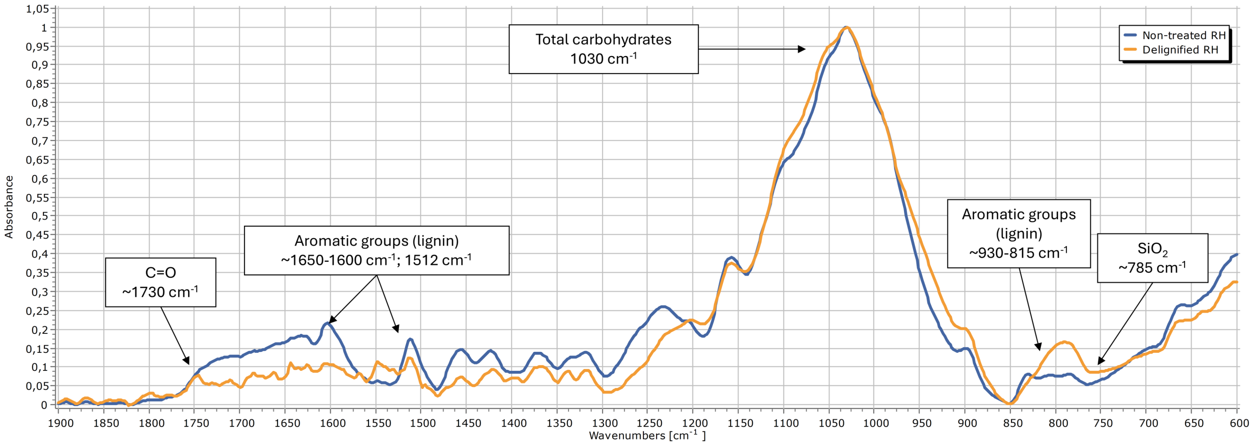


*Figure S10. ATR-FTIR analysis of milled rice husk (particle size 200-400 μm). In blue: raw rice husk; in orange: delignified rice husk.*

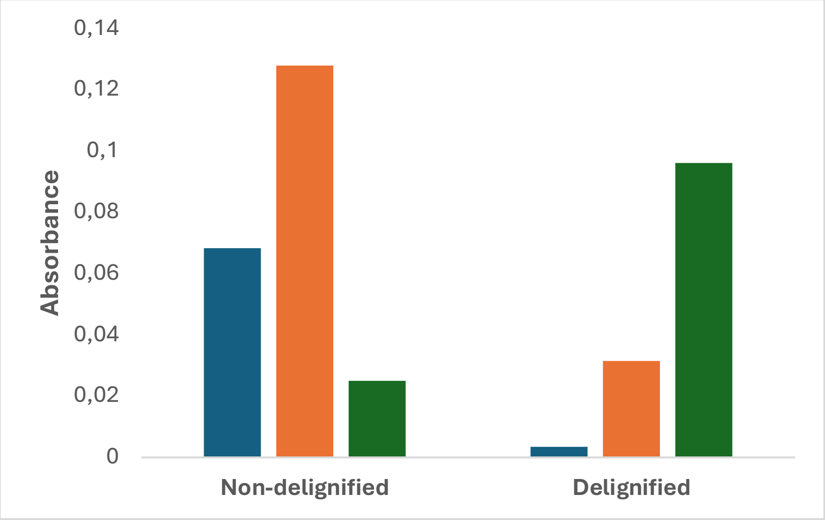


*Figure S11. Variation of the absorbance of the peaks relative to hemicellulose, lignin and cellulose before and after the delignification treatment of rice husk. In blue, the absorbance of hemicellulose (1730 cm$^{-1}$); in orange, the absorbance of lignin (1640 cm$^{-1}$); in green, the absorbance of $SiO_2$ (785 cm$^{-1}$).*

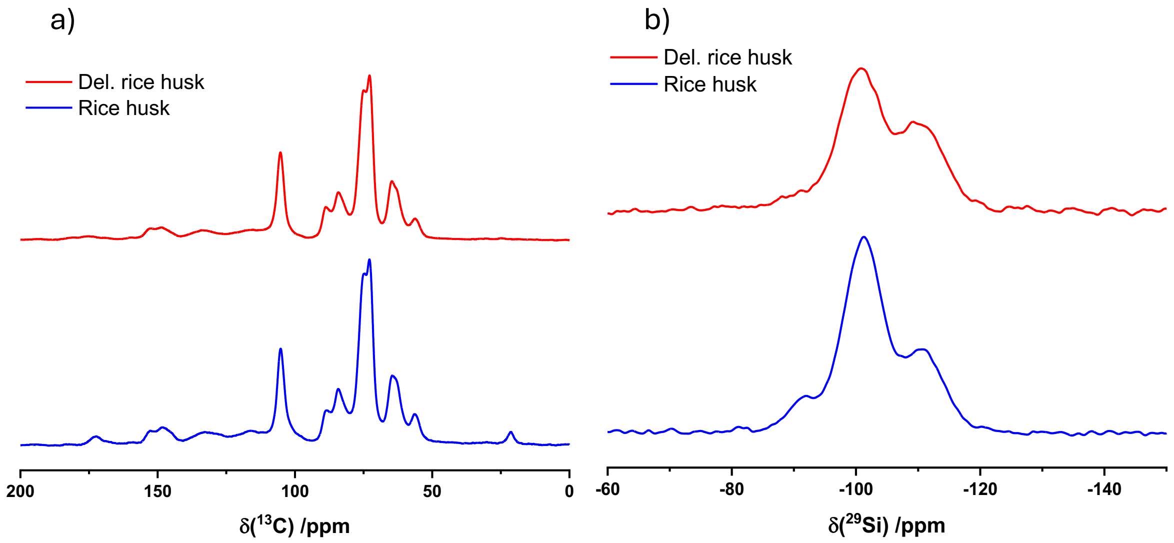


*Figure S12. a) $^{13}C$ CP MAS and b) $^{29}Si$ CP MAS NMR spectra of rice husk (blue trace) and delignified rice husk (red trace).*

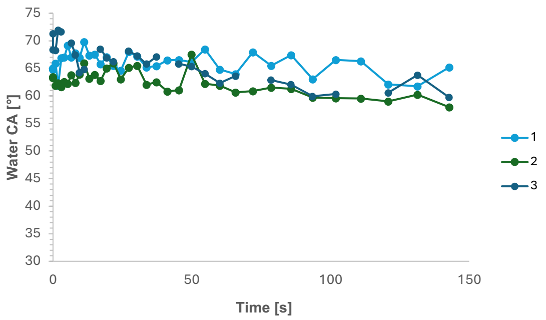


*Figure S13. Static Water Contact Angle evolution in 150 seconds on LA-RH pressed samples. The picture shows 3 repetitions of the measurement after drying the film and redepositing a drop. Volume of the drop: 7 uL.*

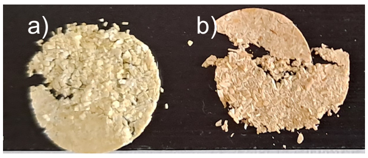


*Figure S14. Pressed samples of d-RH (a) and RH(b) after measurement of water contact angle and drying. The samples are disintegrated.*
